# Essential Role of Cortactin in Myogenic Differentiation: Regulating Actin Dynamics and Myocardin-Related Transcription Factor A-Serum Response Factor (MRTFA-SRF) Signaling

**DOI:** 10.3390/ijms252413564

**Published:** 2024-12-18

**Authors:** Quoc Kiet Ly, Mai Thi Nguyen, Thanh Huu Phan Ngo, Wan Lee

**Affiliations:** 1 Department of Biochemistry, Dongguk University College of Medicine, 123 Dongdae-ro, Gyeongju 38066, Republic of Korea; kietly1501@gmail.com (Q.K.L.); nguyenmainhp@gmail.com (M.T.N.); thngo139@gmail.com (T.H.P.N.); 2 Section of Molecular and Cellular Medicine, Medical Institute of Dongguk University, Dongguk University College of Medicine, 123 Dongdae-ro, Gyeongju 38066, Republic of Korea; 3 Channelopathy Research Center (CRC), Dongguk University College of Medicine, 32 Dongguk-ro, Ilsan Dong-gu, Goyang 10326, Republic of Korea

**Keywords:** cortactin, actin remodeling, myogenic differentiation, MRTFA, SRF, YAP1, mechanotransduction, proliferation

## Abstract

Cortactin (CTTN) is an actin-binding protein regulating actin polymerization and stabilization, which are vital processes for maintaining skeletal muscle homeostasis. Despite the established function of CTTN in actin cytoskeletal dynamics, its role in the myogenic differentiation of progenitor cells remains largely unexplored. In this study, we investigated the role of CTTN in the myogenic differentiation of C2C12 myoblasts by analyzing its effects on actin cytoskeletal remodeling, myocardin-related transcription factor A (MRTFA) nuclear translocation, serum response factor (SRF) activation, expression of myogenic transcription factors, and myotube formation. CTTN expression declined during myogenic differentiation, paralleling the reduction in MyoD, suggesting a potential role in the early stages of myogenesis. We also found that CTTN knockdown in C2C12 myoblasts reduced filamentous actin, enhanced globular actin levels, and inhibited the nuclear translocation of MRTFA, resulting in suppressed SRF activity. This led to the subsequent downregulation of myogenic regulatory factors, such as MyoD and MyoG. Furthermore, CTTN knockdown reduced the nuclear localization of YAP1, a mechanosensitive transcription factor, further supporting its regulatory roles in cell cycle and proliferation. Consequently, CTTN depletion impeded proliferation, differentiation, and myotube formation in C2C12 myoblasts, highlighting its dual role in the coordination of cell cycle regulation and myogenic differentiation of progenitor cells during myogenesis. This study identifies CTTN as an essential regulator of myogenic differentiation via affecting the actin remodeling-MRTFA-SRF signaling axis and cell proliferation.

## 1. Introduction

Skeletal muscle, accounting for approximately half of the body mass, is integral to numerous physiological functions, including locomotion, respiration, metabolism, and energy storage [1]. Myogenesis, the process responsible for muscle tissue development and regeneration, is crucial for maintaining muscle mass, particularly in response to injury or disease [2,3]. This process encompasses myoblast proliferation, differentiation into myocytes, and fusion into multinucleated myotubes, eventually forming functional muscle fibers [4,5]. Myogenesis is regulated by the coordinated interplay of multiple signaling pathways, particularly those controlling cytoskeletal dynamics, which are essential for myoblast differentiation, fusion, and maturation [1,6]. Recent advances in cytoskeleton dynamics research have identified mechanotransduction as a crucial regulatory mechanism in skeletal myogenesis, translating mechanical signals into biochemical responses [7,8,9]. Accordingly, the dysregulation of cytoskeletal remodeling has been causally linked to impaired myoblast differentiation, ultimately hindering myotube formation from progenitor cells [10,11,12,13].

Actin is a dynamic molecule that enables precise temporal and spatial cytoskeleton remodeling, supporting the morphological and functional changes essential for myogenesis [8,14]. In this process, numerous actin-binding proteins (ABPs) are indispensable for dynamic actin remodeling, coordinating actin assembly and disassembly [14,15]. Among these, cortactin (CTTN) plays a pivotal role in actin polymerization and branching [16,17,18,19]. CTTN interacts with the Arp2/3 complex to facilitate the formation of branched actin networks that are essential for cell shape, migration, and adhesion [17,18,19]. CTTN also binds to filamentous actin (F-actin) and enhances actin stability by inhibiting filament depolymerization [18,19,20]. Thus, CTTN has been identified as a key regulator of various cellular processes that require rapid alterations in actin structure and dynamics [16,18,19,20]. However, although the regulatory roles of CTTN in actin dynamics are well established, its specific functions in skeletal myogenesis remain largely unexplored.

Myogenic differentiation is meticulously regulated by key myogenic transcription factors, such as MyoD and MyoG, which coordinate the transition from proliferating myoblasts to differentiated myotubes [6]. Increasing evidence suggests that myocardin-related transcription factor A (MRTFA) and serum response factor (SRF) are involved in a broad spectrum of biological functions, including myofibroblast generation, skeletal myogenesis, neural development, and circadian rhythm [21,22,23,24]. The MRTFA-SRF signaling axis is modulated by the balance between F-actin and globular actin (G-actin), thereby linking actin dynamics to the expression of myogenic transcription factors [25,26,27]. MRTFA passively shifts between the cytoplasm and the nucleus in response to the actin polymerization status, and its nuclear localization is necessary for the activation of SRF, which drives the expressions of critical myogenic genes [21,22,23]. Elevated levels of F-actin promote MRTFA nuclear localization, enhancing SRF activation and facilitating myogenesis [28,29]. Given CTTN’s role in actin dynamics, it likely plays a crucial role in regulating the actin-MRTFA-SRF signaling axis during myogenic differentiation, driving the transcriptional programs critical for myogenesis. Nevertheless, this role remains largely unknown.

Muscle-wasting conditions, such as sarcopenia, muscular dystrophies, and cachexia, are characterized by impaired myogenic differentiation and reduced muscle regeneration [2,30]. Several studies have shown that levels of CTTN are diminished in various muscle-wasting models, including starvation-induced atrophy model in myotubes [31], aged myoblast progenitors [32], and immobilization-induced atrophy in human skeletal muscle [33]. Therefore, it is suggested that CTTN may play a role in preserving muscle mass, where its reduction could contribute to impaired myogenesis and muscle atrophy. Moreover, chronic inflammation and oxidative stress, common features of muscle-wasting conditions, can destabilize cytoskeletal structures and downregulate critical regulatory proteins, including CTTN [34]. Thus, investigating the role of CTTN during myogenesis could provide valuable insights into the mechanisms underlying muscle-wasting diseases and may reveal potential therapeutic targets for enhancing muscle regeneration.

This study aimed to elucidate the role of CTTN in the myogenic differentiation of progenitor cells by examining its impact on actin cytoskeletal dynamics and key mechanosensitive signaling pathways involved in skeletal muscle homeostasis. Specifically, we investigated how CTTN knockdown affects actin remodeling, MRTFA and YAP1 nuclear localization, and SRF activation to drive transcriptional programs essential for myogenic differentiation, facilitating the transition from myoblast proliferation to myotube formation. This research is the first to establish CTTN as a pivotal regulator of myoblast differentiation and proliferation through its influence on actin organization and mechanosensitive transcriptional programming.

## 2. Results

### 2.1. Cortactin (CTTN) Expression Decreased During Myoblast Differentiation

**Figure 1:**
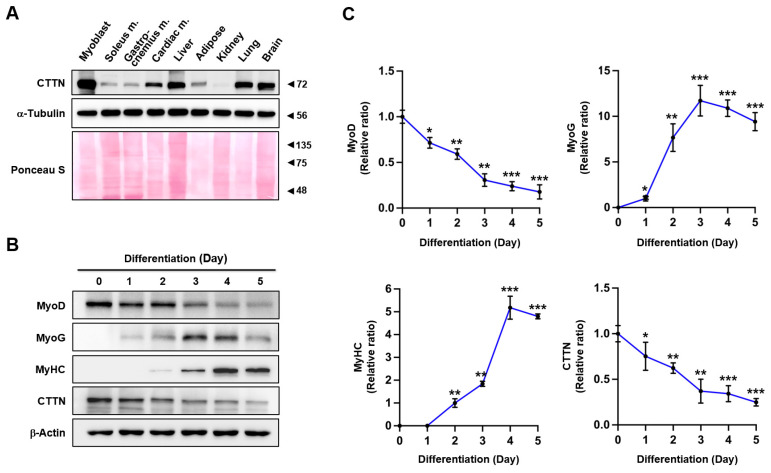
Modulation of CTTN expression during myoblast differentiation. (**A**) Immunoblotting was conducted to assess CTTN expression levels in C2C12 myoblasts and various tissues from C57BL/6 mice, with α-tubulin as a loading control. (**B**) C2C12 myoblasts were harvested on specified differentiation days, and the protein expression levels of MyoD, MyoG, MyHC, and CTTN were analyzed by immunoblotting, with β-actin as a loading control. (**C**) Protein expression levels were normalized to β-actin, and relative expression ratios were calculated, setting day 0 as one for MyoD and CTTN, day 1 for MyoG, and day 2 for MyHC. Data are presented as means ± SEM (n = 3), with asterisks indicating statistical significance (* *p* < 0.05, ** *p* < 0.01, *** *p* < 0.001).

The regulation of differentiation in progenitor cells relies on the actin cytoskeleton remodeling, which is finely orchestrated by ABPs [14,15]. Since CTTN has also been known to regulate actin dynamics, we initially assessed the expressions of CTTN in various mouse tissues, such as C2C12 myoblasts and skeletal and cardiac muscles, before investigating its role in myogenic differentiation. Immunoblot analysis revealed that CTTN is ubiquitously expressed, with notably higher levels in myoblasts than in fully differentiated muscles, including soleus, gastrocnemius, and cardiac muscle (Figure 1A). To explore the role of CTTN in myogenic differentiation further, we determined its expression in C2C12 myoblasts over the 5-day differentiation period (Figure 1B,C). Under our experimental conditions, MyoD, an early marker of myogenic commitment, diminished after the initiation of differentiation. Conversely, MyoG, a key marker of myogenic differentiation onset, increased gradually and peaked on differentiation day 3 before declining. Meanwhile, myosin heavy chain (MyHC), a terminal differentiation marker, gradually increased from day 2, with the highest levels observed from day 4 onwards. Interestingly, the expression patterns of CTTN and MyoD were similar throughout differentiation, with both gradually decreasing as the process progressed toward myotube formation (Figure 1B,C). The similarity in expression patterns between CTTN and MyoD suggests that CTTN might play a significant role in the early stages of myogenic differentiation. We, therefore, postulated that CTTN contributes to this process by modulating the actin cytoskeleton and influencing the expression of myogenic regulatory factors through the mechanosensitive signaling axis.

### 2.2. Cortactin (CTTN) Knockdown Reduced F-Actin and Nuclear Myocardin-Related Transcription Factor A (MRTFA) Levels in Myoblasts

**Figure 2:**
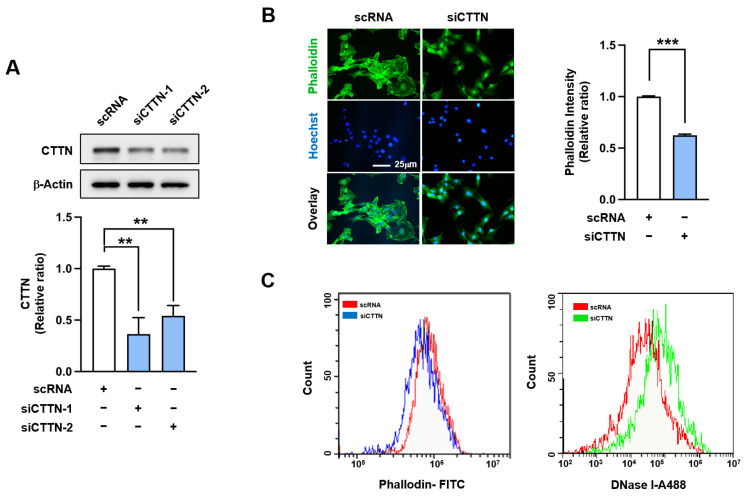
CTTN knockdown led to a reduction in F-actin levels and an increase in G-actin levels. C2C12 myoblasts were transfected with 200 nM of either control scRNA or siCTTN (siCTTN-1 or siCTTN-2). (**A**) CTTN expression was assessed by immunoblotting 24 h after transfection. CTTN expression levels were normalized to β-actin, and relative expression ratios were calculated with the control scRNA set to one. (**B**) After 24 h post-transfection, cells were stained with FITC-phalloidin (green) for F-actin and Hoechst 33,342 (blue) for nuclei. Scale bar: 25 μm. Phalloidin intensities were quantified using ImageJ software, version 1.5.4. (**C**) F- and G-actin levels were quantified by flow cytometry after staining with FITC-phalloidin for F-actin and DNase I for G-actin, respectively. Data are presented as means ± SEM (n = 3), with asterisks indicating statistical significance (** *p* < 0.01, *** *p* < 0.001).

CTTN has been reported to promote actin polymerization by stabilizing F-actin and activating Arp2/3 complex [18,19]. As actin dynamics critically regulate myogenic transcriptional programs [6,35], we hypothesized that the suppression of CTTN would interfere with actin filament assembly, and thereby impair the differentiation of myoblasts. To test this, we transfected C2C12 cells with control scRNA or CTTN-specific siRNAs (siCTTN-1 or siCTTN-2) and cultured them for 24 h in GM. Transfection with siCTTN-1 or siCTTN-2 resulted in a 50~60% reduction in CTTN protein levels compared to scRNA controls (Figure 2A). Although siCTTN-2 also exhibited the myogenic regulatory effect similar to siCTTN-1 (Supplementary Figures S2–S8), we chose to use siCTTN-1 (hereafter referred to as siCTTN) in subsequent experiments due to its more robust and effective suppression of CTTN protein levels.

Next, we evaluated whether CTTN knockdown using siCTTN affected the distribution between F-actin and G-actin in C2C12 myoblasts. As expected, siCTTN significantly reduced F-actin levels compared to scRNA controls, as determined by F-actin staining using FITC-phalloidin (Figure 2B). Flow cytometry verified that CTTN knockdown decreased F-actin levels but increased G-actin levels, suggesting enhanced actin cytoskeletal depolymerization (Figure 2C). Since total actin levels remained unchanged after transfections, this decrease in F-actin was attributed to impaired actin polymerization resulting from CTTN suppression.

**Figure 3:**
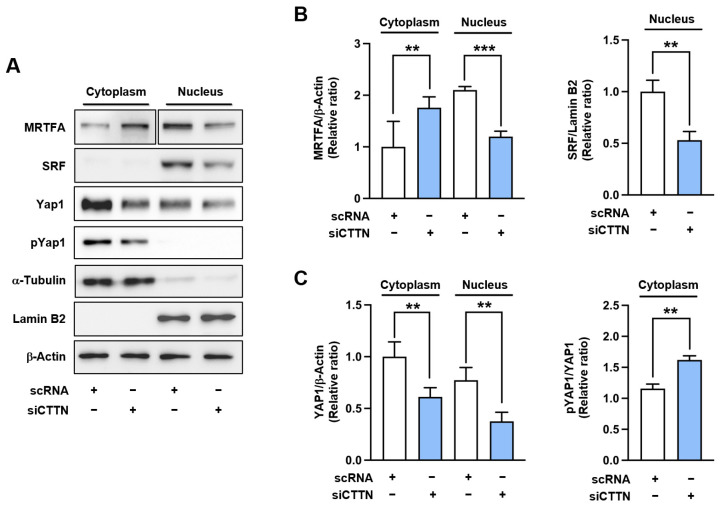
CTTN depletion impaired the nuclear localization of MRTFA and YAP1. C2C12 myoblasts were transfected with either control scRNA or siCTTN and analyzed 24 h post-transfection. (**A**) Cytoplasmic and nuclear fractions were subjected to immunoblot analysis for MRTFA, SRF, YAP1, pYAP1 (phosphorylated YAP1), and CTTN expression. For MRTFA, different exposure times were used to account for its varied distribution between cytoplasmic and nuclear compartments. α-Tubulin and lamin B2 served as cytoplasmic and nuclear markers, respectively. β-Actin was used as a loading control. (**B**,**C**) The protein expression levels were normalized to β-actin, and relative expression ratios were calculated with the control scRNA set to one. Data are presented as means ± SEM (n = 3), with asterisks indicating statistical significance (** *p* < 0.01, *** *p* < 0.001).

MRTFA is a coactivator of SRF, and actin dynamics regulate its nuclear translocation [21,25,28]. Given the association between G-actin accumulation and MRTFA retention in the cytosol, we next investigated whether CTTN knockdown would increase cytosolic MRTFA levels and inhibit its nuclear translocation. As shown in Figure 3A,B, transfecting C2C12 myoblasts with scRNA or siCTTN revealed that CTTN knockdown significantly increased cytoplasmic MRTFA levels and concomitantly decreased its nuclear levels, indicating the inhibition of MRTFA nuclear translocation. Moreover, CTTN knockdown markedly decreased the levels of SRF protein in the nucleus (Figure 3A,B). Thus, our data indicate that CTTN suppression inhibits actin polymerization, increases cytoplasmic G-actin levels, and subsequently inhibits MRTFA nuclear translocation and SRF expression.

Inhibition of actin polymerization has been linked to decreased nuclear translocation of YAP1, a mechanosensitive transcription factor that promotes cell proliferation, by enhancing its phosphorylation and degradation [36,37]. To assess whether CTTN knockdown also affects YAP1 localization, we measured the phosphorylation and expression levels of YAP1 in both the cytoplasmic and nuclear fractions of C2C12 myoblasts. Our results showed significant decreases in cytoplasmic and nuclear YAP1 levels by siCTTN transfection (Figure 3A,C). These findings indicate that CTTN knockdown reduces F-actin accumulation, which in turn increases YAP1 phosphorylation and suppresses its nuclear localization.

### 2.3. Cortactin (CTTN) Depletion Inhibited Serum Response Factor (SRF) Promoter Activity and the Expressions of Genes Targeted by Serum Response Factor (SRF)

**Figure 4:**
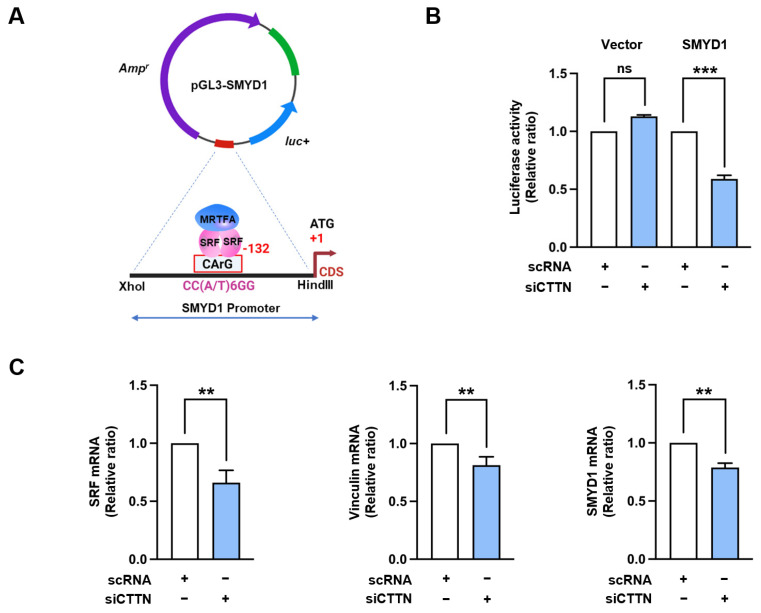
CTTN knockdown suppressed SRF transcriptional activity. (**A**) Diagram of the luciferase reporter construct featuring the truncated SMYD1 promoter region, including the CArG box for SRF binding. (**B**) C2C12 myoblasts were transfected with either the pGL3 vector (Vector) or pGL3 containing the SMYD1 promoter (SMYD1) along with control scRNA or siCTTN. Relative luciferase activity was measured 24 h post-transfection. (**C**) C2C12 myoblasts were transfected with either control scRNA or siCTTN, and mRNA levels of SRF, Vinculin, and SMYD1 were assessed by RT-qPCR, normalized to GAPDH expression 24 h post-transfection. Data are presented as means ± SEM (n = 3), with asterisks indicating statistical significance (** *p* < 0.01, *** *p* < 0.001); ns indicates non-significance.

SMYD1 is a histone-lysine N-methyltransferase specific to skeletal muscle and serves as a target gene of the MRTFA-SRF regulatory pathway during skeletal muscle differentiation [38,39]. Hence, to investigate the effect of CTTN depletion on SRF activation, we subcloned the SRF target site (CArG box) within the SMYD1 gene promoter into the pGL3 dual luciferase reporter vector as illustrated in Figure 4A. Following this, luciferase activity was measured in C2C12 myoblasts after transfection with either the control vector (pGL3) or the pGL3-SMYD1 reporter vector, along with either scRNA or siCTTN. Indeed, CTTN knockdown significantly reduced luciferase activity by ~45% compared to scRNA controls (Figure 4B), suggesting impaired SRF promoter activity. RT-*q*PCR analysis revealed that transcripts of SRF target genes, including those of vinculin, SMYD1, and SRF itself, were substantially decreased in myoblasts transfected with siCTTN (Figure 4C). These results suggest that CTTN depletion inhibits SRF-mediated transcription, highlighting the importance of CTTN in the regulation of SRF activity in myoblasts.

### 2.4. Cortactin (CTTN) Knockdown Impeded Myoblast Proliferation

**Figure 5:**
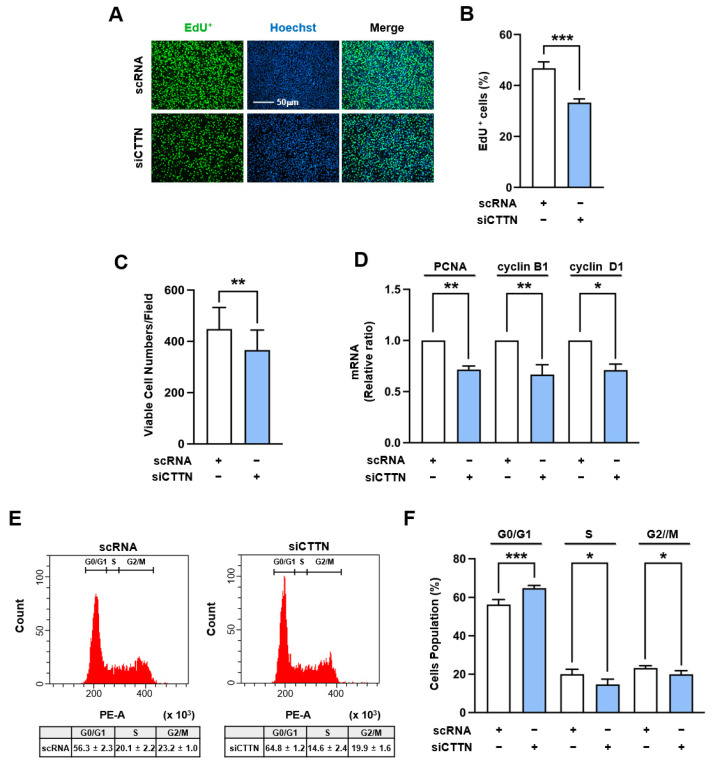
CTTN depletion impeded cell proliferation and cell cycle progression. C2C12 myoblasts were transfected with either control scRNA or siCTTN and analyzed 24 h post-transfection. (**A**) Cell proliferation was evaluated by EdU incorporation (green) to label replicating cells, with Hoechst 33,342 (blue) as a nuclear counterstain. Scale bar: 50 µm. (**B**) The percentage of EdU-positive cells was quantified using ImageJ software. (**C**) Viable cell numbers were measured using a cell viability assay kit. (**D**) mRNA levels of proliferation markers (PCNA, cyclin B1, and cyclin D1) were assessed by RT-*q*PCR and normalized to GAPDH expression. (**E**,**F**) Cell cycle analysis was performed using flow cytometry with scatter plots. Data are presented as means ± SEM (n = 3), with asterisks indicating statistical significance (* *p* < 0.05, ** *p* < 0.01, *** *p* < 0.001).

Since nuclear MRTFA and YAP1 levels, which have been known to promote cell cycle progression and proliferation [40,41,42,43,44], were significantly reduced by CTTN knockdown, we further determined whether CTTN knockdown affects cell proliferation and cell cycle progression after transfecting C2C12 cells with siCTTN. EdU-coupled fluorescence intensity analysis and viable cell counting revealed significant reductions in both the proportion of EdU-positive cells and total cell numbers in siCTTN-transfected myoblasts (Figure 5A–C). Since siCTTN did not induce apoptosis or necrosis in myoblasts (Supplementary Figure S1), the observed reduction in EdU incorporation and cell viability can be attributed to inhibited proliferation, suggesting that these myoblasts exhibited a reduced proliferative capacity compared to the controls. In addition, RT-qPCR showed that the mRNA levels of proliferating cell nuclear antigen (PCNA), cyclin B1, and cyclin D1, which are target genes of the MRTFA-SRF axis and YAP1 signaling related to cell proliferation, were significantly downregulated in C2C12 myoblasts transfected with siCTTN (Figure 5D). Moreover, flow cytometry supported these findings by revealing a decrease in the percentage of cells in the S and G2/M phases, along with an increase in the proportion of cells in the G0/G1 phase (Figure 5E,F). Thus, these results indicate that the depletion of CTTN inhibits both cell proliferation and cell cycle progression in myoblasts.

### 2.5. Cortactin (CTTN) Is Required for Myogenic Differentiation

**Figure 6:**
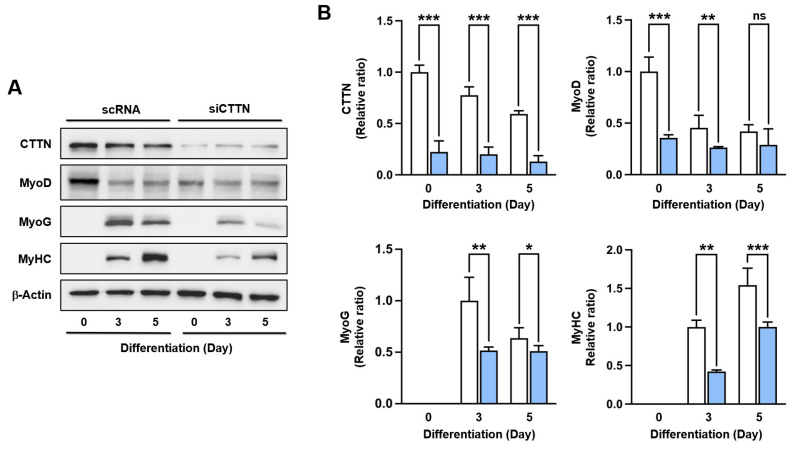
CTTN knockdown suppressed the expression of myogenic regulatory factors. (**A**) C2C12 myoblasts were transfected with either control scRNA or siCTTN, allowed to differentiate, and then harvested on specified differentiation days. Protein expression levels of MyoD, MyoG, MyHC, and CTTN were analyzed by immunoblotting. (**B**) Protein expression levels for scRNA (open column) and siCTTN (blue column) were normalized to β-actin and presented as relative ratios, with scRNA expression levels on day 0 (for CTTN and MyoD) or day 3 (for MyoG and MyHC) set to one. Data are presented as means ± SEM (n = 3), with asterisks indicating statistical significance (* *p* < 0.05, ** *p* < 0.01, *** *p* < 0.001); ns indicates non-significance.

Since CTTN knockdown inhibited the actin-MRTFA-SRF axis, YAP1 nuclear localization, and cell cycle progression, we next examined its effect on myogenic differentiation. We determined the protein expression of MyoD, MyoG, and MyHC on differentiation days 0, 3, and 5 following transfections with either scRNA or siCTTN. Our results indicated that MyoD, MyoG, and MyHC protein levels were significantly lower in siCTTN-transfected myoblasts compared to scRNA controls (Figure 6A,B). MyoD, a target gene of the actin-MRTFA-SRF signaling axis, initiates the differentiation process by triggering the expression of MyoG, which in turn drives myoblast differentiation [39,45,46]. Consequently, the levels of MyoG and MyHC were notably reduced following CTTN knockdown, suggesting an impaired expression of myogenic regulatory factors. These findings demonstrate that CTTN is crucial for initiating the expression of myogenic transcription factors, including MyoD and MyoG, through the actin-MRTFA-SRF axis.

**Figure 7:**
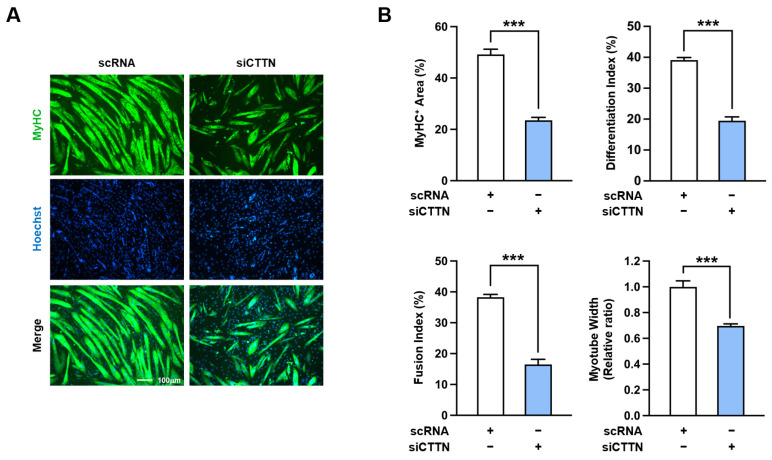
CTTN depletion impaired myogenic differentiation. C2C12 myoblasts were transfected with either control scRNA or siCTTN and then allowed to differentiate for 5 days. (**A**) Representative immunocytochemistry stained with MyHC antibody (green) and Hoechst 33,342 (blue). Scale bar: 100 μm. (**B**) MyHC-positive areas, differentiation indices, fusion indices, and myotube widths were determined as described in Section 4. Data are presented as means ± SEM (n = 3), with asterisks indicating statistical significance (*** *p* < 0.001).

To further confirm the role of CTTN in myoblast differentiation and myotube formation, we performed immunocytochemical analysis in C2C12 cells using MyHC antibody after five days of differentiation. As shown in Figure 7A,B, siCTTN-transfected myoblasts exhibited significantly less differentiation and myotube formation than scRNA controls, as evidenced by lower MyHC-positive area, differentiation index, fusion index, and myotube width. Collectively, these findings demonstrate that CTTN is crucial for initiating myogenic transcription programs, supporting the expression of myogenic regulatory factors, including MyoD and MyoG, and ensuring the proper differentiation and myotube formation of progenitor cells.

## 3. Discussion

Actin dynamics are crucial in the regulation of myogenesis by linking mechanosensitive transcription programs to the expression of myogenic genes and modulating the cell cycle, all of which are essential for the precise coordination of myogenic processes [7,8,9]. Although growing evidence indicates that CTTN promotes actin polymerization and stabilizes actin filaments [17,47], its functional significance in myogenic differentiation has not been investigated. The findings of this study provide novel insights into the function of CTTN as a pivotal regulator of myogenic differentiation by modulating the actin-MRTFA-SRF signaling pathway and myoblast proliferation as follows: (i) CTTN knockdown increases cytoplasmic G-actin levels and impairs the nuclear translocation of MRTFA, resulting in diminished SRF activity and downregulation of myogenic regulatory factors, such as MyoD and MyoG. (ii) CTTN knockdown reduced the nuclear levels of MRTFA and YAP1, further highlighting its dual role in the coordination of proliferation and differentiation of progenitor cells during myogenesis. (iii) Consequently, CTTN depletion impedes myogenic differentiation and myotube formation of progenitor cells.

Our findings suggest that CTTN supports myogenic commitment during the early stages by stabilizing F-actin and facilitating MRTFA-SRF-driven MyoD activation, which is necessary for MyoG expression and differentiation. By stabilizing F-actin, CTTN promotes the formation of branched actin networks, which are essential for providing structural support and facilitating signaling pathways that drive effective myoblast differentiation [48]. We propose that although CTTN levels decrease as differentiation proceeds, maintaining a balanced expression in the early stages is essential to stabilize F-actin and uphold key myogenic signals. This regulated decline allows the cytoskeleton to remain adaptable, preventing over-stabilization that could hinder muscle maturation. Thus, we speculate that CTTN serves a regulatory function throughout myogenesis, where its knockdown disrupts critical MRTFA-SRF and YAP1 signaling, leading to reduced proliferation and impaired differentiation. Consequently, controlled CTTN expression appears essential for proper muscle development.

Indeed, CTTN depletion led to a reduction in F-actin levels and an increase in G-actin levels in myoblasts. Although we could not directly assess actin polymerization kinetics and stability in this study, the observed shift is likely due to the inhibition of primary CTTN functions, leading to actin depolymerization and filament destabilization following its knockdown. It has been well-established that actin cytoskeletal remodeling is essential for the nuclear translocation of MRTFA, which acts as a coactivator of SRF and drives the expressions of key myogenic regulatory genes, including MyoD and MyoG [25,26,27]. In this aspect, the disruption of actin remodeling caused by CTTN depletion hindered the nuclear translocation of MRTFA, which inhibited SRF activation and led to the downregulation of the expressions of myogenic genes. These findings underscore the importance of CTTN in maintaining actin cytoskeletal integrity and enabling efficient MRTFA-SRF signaling during myogenesis [26,28].

Our analysis of SRF activity further emphasized the integral role played by CTTN in the regulation of myogenic gene expressions. CTTN knockdown diminished the transcriptional activity of SRF, as demonstrated by decreased luciferase activity using SMYD1 promoter. Consequently, the expression of SRF target genes, such as MyoD, SMYD1, vinculin, and SRF itself, were significantly reduced. MyoD is a critical regulator of myogenic commitment and essential for initiating MyoG expression [46]. Therefore, the observed reduction in MyoG expression in CTTN-depleted cells was attributed to decreased MyoD levels, which were directly influenced by impaired SRF activity. Furthermore, the reduced expression of SMYD1, a key promoter of myotube formation, further highlights the impact of CTTN depletion on muscle differentiation. Given that SMYD1 is crucial for myogenic differentiation and myotube formation [39] and its downregulation is associated with impaired differentiation and myofibrillar disorganization [39,49], our findings indicate that CTTN is a key regulator of myogenesis.

CTTN also plays a critical role in myoblast proliferation. We observed that CTTN knockdown significantly impaired myoblast proliferation, as evidenced by reduced cell viability, decreased EdU incorporation, and downregulation of cell cycle-related genes (PCNA, cyclin B1, and cyclin D1). In addition, flow cytometry revealed the cell cycle arrest in the G0/G1 phase, suggesting impaired cell cycle progression. Since progenitor cell proliferation is a prerequisite for myogenesis and the MRTFA-SRF axis has been implicated in the regulation of cell cycle progression [40,41,42], these findings align with the MRTFA-SRF axis’s role in activating genes essential for cell proliferation [42,50]. Moreover, increasing evidence suggests that MRTFA and SRF promote oncogenesis by inducing cell proliferation, epithelial-to-mesenchymal transition, and metastasis [42,51,52,53]. These findings support the notion that CTTN is involved in cell cycle and proliferation regulation via the actin-MRTFA-SRF pathway during myoblast differentiation. In addition to the actin-MRTFA-SRF axis, the suppression of cell proliferation resulting from CTTN knockdown is linked to the inactivation of mechanosensitive transcriptional coactivator YAP1, which is known to respond to cytoskeletal tension and regulate cell proliferation [43,44]. In our study, CTTN depletion reduced nuclear YAP1 levels by enhancing YAP1 phosphorylation in the cytoplasm, leading to its degradation and reduced nuclear accumulation. This suggests that CTTN is essential for maintaining the mechanical cues necessary for proper YAP1 signaling, which promotes cell proliferation and survival. The impaired balance between proliferation and differentiation observed in CTTN-depleted myoblasts underscores the importance of the coordinating role of CTTN in these processes during myogenesis, as this balance is crucial for effective muscle development and regeneration.

The present study showed that the regulatory roles of CTTN during differentiation and proliferation are due to its effects on cytoskeletal dynamics and mechanotransduction pathways. During myogenesis, progenitor cells must exit the cell cycle and undergo differentiation to form mature muscle fibers. CTTN ensures proper actin polymerization, which is necessary for activating the MRTFA-SRF and YAP1 signaling pathways. This dual regulation underscores the complexity of the signaling networks that control muscle cell fate decisions and emphasizes the importance of actin cytoskeletal remodeling in determining the balance between proliferation and differentiation. Thus, the disruption of this balance, as observed in CTTN-depleted cells, might impair muscle regeneration and contribute to muscle-wasting conditions.

The implications of our findings are particularly significant for muscle-wasting diseases, such as sarcopenia and muscular dystrophy. Reduced levels of CTTN have been observed in various muscle-wasting conditions, including atrophy caused by starvation in myotubes [31], age-related changes in myoblast progenitors [32], and immobilization-induced atrophy in human skeletal muscle [33]. Our study provides mechanistic insights into how CTTN depletion may contribute to muscle-wasting diseases by impairing the integrity of the actin cytoskeleton and disrupting MRTFA-SRF signaling, ultimately leading to defective muscle development and regeneration. These findings emphasize the importance of maintaining cytoskeletal stability for muscle health.

**Figure 8:**
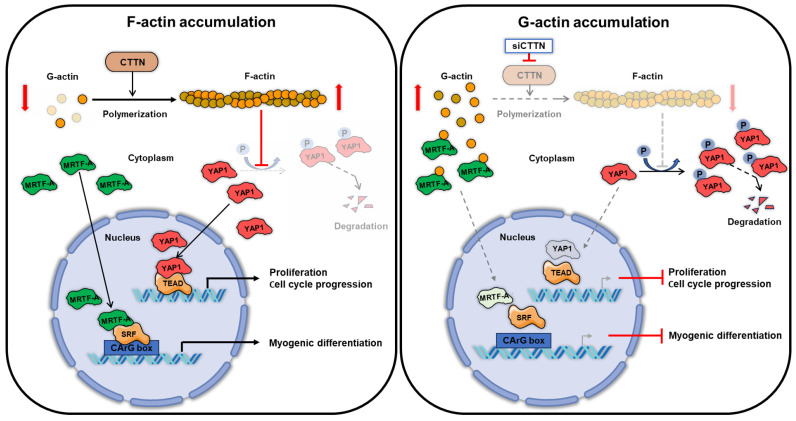
Schematic illustration of the actin-MRTFA-SRF and YAP1 signaling pathway regulated by CTTN.

As illustrated in Figure 8, the present study identifies CTTN as a pivotal regulator of myoblast differentiation and proliferation through its influence on actin cytoskeletal dynamics and the mechanosensitive transcription program for myogenic differentiation. CTTN depletion leads to actin depolymerization, the impaired nuclear localizations of MRTFA and YAP1, reduced SRF activation, and consequently provokes the downregulation of key myogenic genes to inhibit myotube formation and myoblast proliferation. This study provides valuable insights into the molecular mechanisms underlying skeletal myogenesis and identifies CTTN as a promising therapeutic target for muscle-related disorders. Further investigations on the regulation and function of CTTN would enhance our understanding of muscle biology and possibly lead to novel treatments for muscle degeneration and related conditions.

## 4. Materials and Methods

### 4.1. Cell Culture

C2C12 cells (a myoblast cell line) were purchased from the ATCC (CRL-1772, Manassas, VA, USA) and grown in a medium consisting of DMEM, 10% Fetal Bovine Serum (FBS), and 100 units/mL penicillin/streptomycin (Gibco, Carlsbad, CA, USA), hereafter referred to as growth medium (GM). To induce differentiation, C2C12 cells were plated on 35 mm dishes (~1.3 × 10⁵ cells/dish) in GM. When cells were ~90% confluent, differentiation was induced by switching to a differentiation medium (DM; DMEM containing 2% horse serum (Gibco)) and incubating cells for up to five days. The medium was replaced with fresh DM every 24 h.

### 4.2. Oligonucleotide Transfection

C2C12 cells were grown in 35 mm dishes at ~1.3 × 10⁵ cells per dish for 20–24 h until 40–50% confluent. Cells were then transfected with 200 nM scrambled control RNA (scRNA) (Genolution, Seoul, Republic of Korea), CTTN siRNA (siCTTN-1, Bioneer, Daejeon, Republic of Korea), or another CTTN siRNA (siCTTN-2, Genolution, Seoul, Republic of Korea) using Lipofectamine 2000 (Invitrogen, Thermo Fisher Scientific, Waltham, MA, USA). Transfected cells were incubated in serum-free DMEM for 4 h and then maintained in GM for 24 h. Oligonucleotide sequences are presented in Table S1.

### 4.3. Reverse Transcription Quantitative Polymerase Chain Reaction (RT-qPCR)

Total RNA was obtained using a Total RNA Miniprep kit (Enzynomics, Daejeon, Republic of Korea), and total RNA concentrations were determined using a micro-volume spectrophotometer (Nanodrop, Keen Innovative Solutions, Daejeon, Republic of Korea). Complementary DNA was synthesized from RNA using an miScript II RT Kit (Qiagen, Hilden, Germany). Relative mRNA expressions were determined by RT-qPCR using SYBR Green (Enzynomics, Daejeon, Republic of Korea). RT-qPCR assays were conducted in triplicate. The expression levels were normalized versus GAPDH using the 2^–ΔΔCt^ method. The primer sequences and conditions applied in this study are provided in Table S2.

### 4.4. Dual-Luciferase Assay

The promoter region of SMYD1, containing the CC(A/T)_6_GG sequence (CArG box), was cloned into the pGL3-Basic reporter vector (Promega, Madison, WI, USA) to create the pGL3-SMYD1 reporter construct. The oligonucleotide sequences used for cloning are listed in Table S3. For the dual-luciferase assay, C2C12 cells were seeded in 12-well plates and co-transfected with the pRLSV40P vector (50 ng, encoding Renilla luciferase as an internal control) and the pGL3-SMYD1 reporter vector (50 ng) along with either scrambled RNA (scRNA) or siCTTN, using Lipofectamine 2000 (Invitrogen). Transfections were performed in DMEM without serum or antibiotics. After 6 h, the transfection medium was replaced with growth medium (GM), and cells were harvested 24 h later. Luciferase activities were measured using the Dual-Luciferase Reporter Assay System (Promega) following the manufacturer’s instructions. The pGL3-Basic vector, which lacks a promoter, ensured no basal expression of luciferase. The SMYD1 promoter sequence inserted into the vector enabled a specific measurement of promoter-driven luciferase activity. Experimental results were normalized to untreated control samples, which were assigned a value of 1, to assess the impact of treatments. The Renilla luciferase activity from the co-transfected pRLSV40P vector was used to normalize transfection efficiency and account for background signals.

### 4.5. Cytoplasmic and Nuclear Fraction Extraction

After 24 h of transfection, C2C12 cells were harvested using trypsin/EDTA solution (Gibco). According to the manufacturer’s manual, cytoplasmic and nuclear fractions were isolated using NE-PER Reagents (Thermo Fisher Scientific). Briefly, pellets were incubated on ice for 10 min with CER I solution; CER II solution was applied at the last minute. Cell lysates were centrifuged for 15 min at ~15,000 rpm at 4 °C, and supernatants (cytoplasmic fractions) were transferred to clean pre-chilled tubes. Residues were suspended in NER solution to extract nuclear fractions. An equal amount of cytoplasmic and nuclear fractions was used for immunoblot analysis.

### 4.6. Immunoblot Analysis

Total protein was extracted using a lysis buffer composed of 1% phosphatase inhibitor cocktail II, 0.2 mM PMSF, and 2% Triton-X in PBS (Sigma-Aldrich, St. Louis, MO, USA). Protein concentrations were measured using the Bradford assay, and samples were denatured at 100 °C in sample buffer for 10 min. Equal amounts of protein (20 µg) were separated by SDS-PAGE and transferred to nitrocellulose membranes (Amersham Biosciences, Piscataway, NJ, USA). The membranes were blocked with 5% skimmed milk (Difco) in TBST solution (0.5% TBS-Tween 20) and incubated overnight at 4 °C with primary antibody. The following day, the membranes were washed 6 times for 5 min with TBST and then incubated with secondary antibody diluted at 1:10,000. Blots were developed using TOPview^TM^ ECL Femto Western Substrate (Enzynomics, Daejeon, Republic of Korea) and analyzed with a protein band density measurement software package (Fusion Solo, Paris, France). The antibodies used in this study are listed in Table S4.

### 4.7. Immunocytochemistry

Differentiated C2C12 cells were fixed in 4% paraformaldehyde for 10 min, permeabilized with 0.3% Triton X-100 for 15 min, and blocked in 3% heat-inactivated bovine serum albumin in PBS for 2 h at room temperature. Immunocytochemistry was conducted by incubating samples with anti-myosin heavy chain (MyHC) antibody at a 1:100 dilution overnight at 4 °C. Samples were then washed with PBS, incubated with an Alexa 488-conjugated secondary antibody (Invitrogen) for 1 h at room temperature, and counterstained with Hoechst 33,342 (Invitrogen) to visualize cell nuclei. Fluorescent images were captured from five randomly selected fields using a Leica fluorescence microscope (Microsystems, Mannheim, Germany). Experiments were performed in at least three independent replicates. MyHC-positive areas, nucleus numbers in myotubes, and myotube widths were determined using ImageJ software, and differentiation and fusion indexes were calculated as previously described [12]. Briefly, differentiation indices were determined by calculating the ratio of nuclei positive for MyHC within myotubes to the total nuclei count in a given field. Fusion indices were assessed by quantifying the proportion of myotubes containing three or more nuclei, expressed as a percentage of the total nuclei present. For each parameter, a minimum of five randomly selected regions were analyzed across three independent cultures to ensure statistical robustness. F-actin was stained using FITC-conjugated phalloidin (Sigma). Fluorescence intensity was measured using ImageJ software. The total fluorescence, such as MyHC area and phalloidin intensity, within each field, was quantified and normalized to the number of Hoechst-positive nuclei to account for variations in cell number across images.

### 4.8. Cell Proliferation Assay

C2C12 cells were seeded in an 8-chamber slide at a density of 3 × 10^4^ cells/well and transiently transfected. Then, 24 h post-transfection, cells were incubated with 10 µM EdU for 4 h at 37 °C, fixed with 4% PFA, and permeabilized with 0.3% Triton X-100. A 300 µL Click-iT reaction cocktail was added, and cells were then counterstained with Hoechst 33342 for 15 min. Fluorescent images were captured using a Leica fluorescence microscope. To determine the proportion of EdU-positive cells, total and EdU-positive cell numbers were counted in five randomly selected images. Experiments were performed in at least three independent replicates.

### 4.9. Cell Viability

Myoblasts were seeded in 96-well plates at 10^3^ cells per well, transfected with the indicated small interfering RNA using Lipofectamine 2000 (Invitrogen). After 24 h, cells were cultured in GM containing 10 µL of Quanti-Max WST-8 Cell Viability Assay Kit solution (BioMax, Seoul, Republic of Korea) for 4 h at 37 °C. Cell viability was measured at 450 nm using a microplate reader (Model 680, Bio-Rad, Hercules, CA, USA).

### 4.10. Flow Cytometry

Myoblasts were collected using trypsin-EDTA (Gibco), centrifuged at 5000 rpm for 5 min at 4 °C, and rinsed with PBS buffer. After discarding the supernatants, cells were fixed overnight in 70% ethanol at 4 °C. Cell suspensions were then stained for 20 min in the dark using a Propidium Iodide Flow Cytometry Kit (ab139418, Abcam, Cambridge, UK) for cell cycle analysis, FITC-phalloidin for F-actin measurement, or DNase I for G-actin measurement. Samples were analyzed on a CytoFLEX instrument (Beckman Coulter, Brea, CA, USA).

### 4.11. Statistical Analysis

Analysis was performed using one-way ANOVA with a post hoc test for multiple comparisons. Results are shown as means ± standard errors (SEM) from at least three independent experiments.

## Data Availability

The data presented in this study are available on request from the corresponding author.

## Supplementary Materials

The following supporting information can be downloaded at: https://www.mdpi.com/article/10.3390/ijms252413564/s1.
