# Supplementary material for: Essential Role of Cortactin in Myogenic Differentiation: Regulating Actin Dynamics and Myocardin-Related Transcription Factor A-Serum Response Factor (MRTFA-SRF) Signaling
Source: Int J Mol Sci. 2024 Dec 18;25(24):13564. doi: 10.3390/ijms252413564 (PMC11677934; doi:10.3390/ijms252413564)
Supplement: Supplementary file 1 [file ijms-25-13564-s001.zip › Supplementary Figures.pdf]

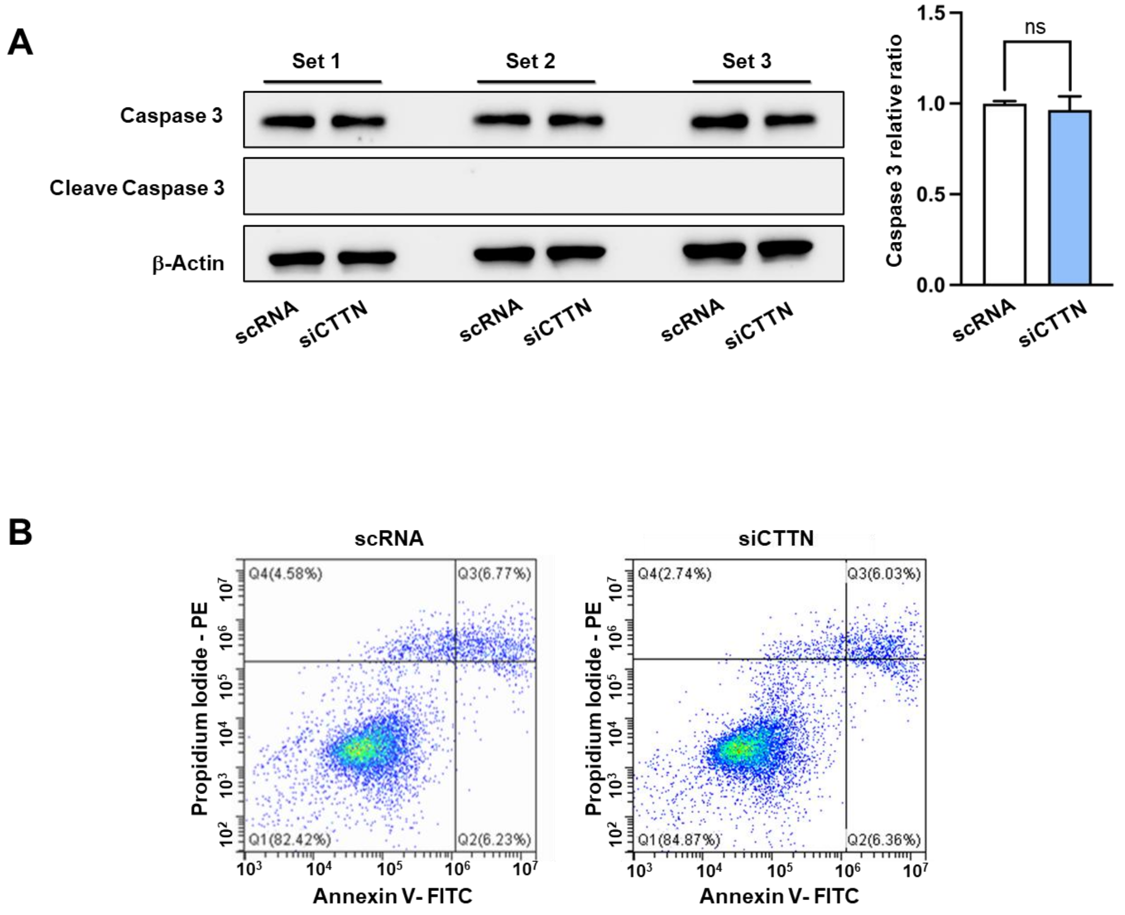

Figure S1. C2C12 cells were transfected with scRNA or siCTTN for 24 hrs. (**A**) Immunoblot analysis of caspase-3 expression to evaluate apoptosis. Total caspase-3 and cleaved caspase-3 were detected using the caspase-3 antibody (Cell Signaling Technology, #9662). β-Actin was used as a loading control. (**B**) Flow cytometry analysis of apoptosis was performed using the FITC Annexin V Apoptosis Detection I Kit (BD Pharmingen). Annexin V and PI staining identified apoptotic (Annexin V+/PI− for early apoptosis, Annexin V+/PI+ for late apoptosis) and necrotic cells.

Apoptosis assay

The apoptosis assay was performed using Flow cytometry with FITC Annexin V Apoptosis Detection I (BD Pharmingen, California, USA). Briefly, after transfection, cells (10^5 cells) were harvested using Trypsin-EDTA, centrifuged, and washed twice with PBS. The cell pellets were then resuspended in 100uL 1X Binding buffer in an e-tube. 5 μL of FITC Annexin V and 5 μL PI were added, and the cells were gently vortexed. The samples were incubated for 15 min at room temperature, protected from light. Finally, 400 μL of 1X Binding Buffer was added to each tube, and the samples were analyzed using a CytoFLEX flow cytometer (Beckman Coulter, USA) and CytoFLEX software.

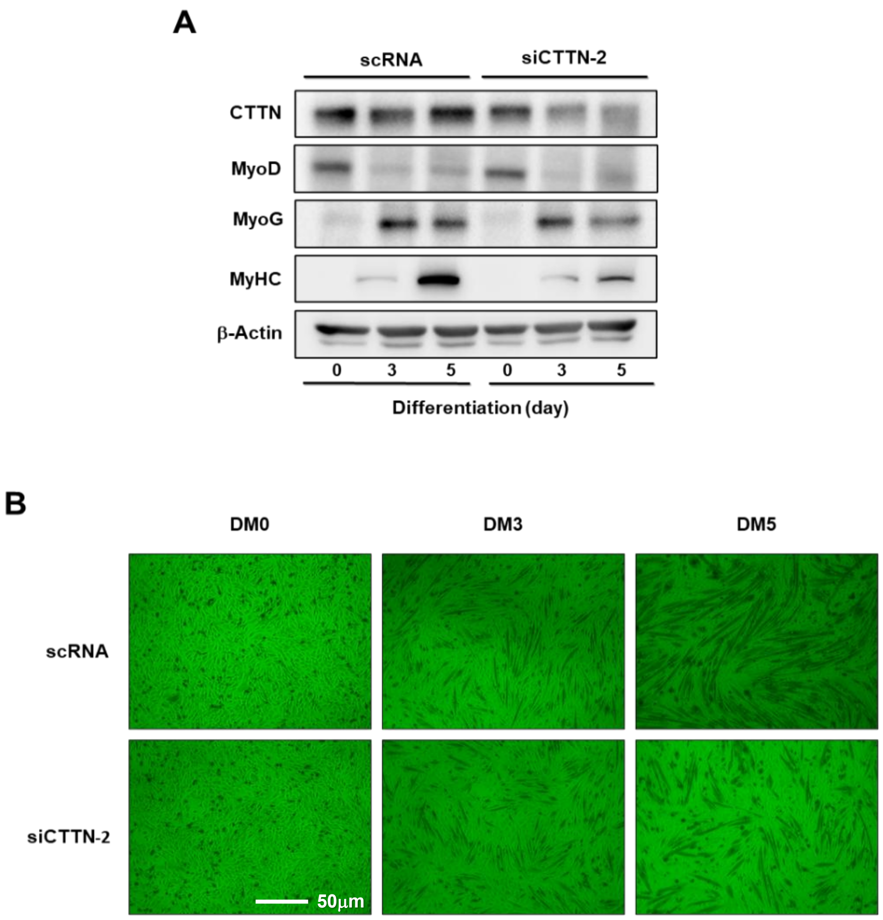

Figure S2. siCTTN-2 suppressed the expression of myogenic genes and inhibited myogenic differentiation. C2C12 cells were transfected with scRNA or siCTTN-2 and allowed to differentiate for up to 5 days. (**A**) Immunoblot analysis of myogenic markers (MyoD, MyoG, and MyHC) and cortactin (CTTN) during differentiation. Protein expression was evaluated at days 0, 3, and 5 of differentiation using specific antibodies. β-Actin was used as a loading control. (**B**) Phase-contrast microscopy images of myotube formation were captured at 0, 3, and 5 days post-differentiation using a Leica microscope. Scale bar: 50 µm. Representative images demonstrate reduced myotube formation in siCTTN-2-transfected cells compared to scRNA-transfected controls.

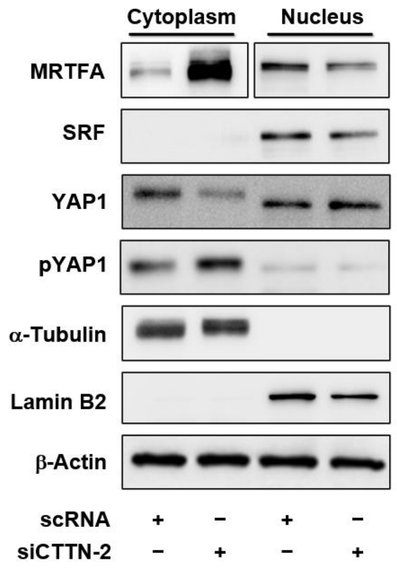

Figure S3. siCTTN-2 impaired the nuclear localization of MRTFA and YAP1. C2C12 myoblasts were transfected with either control scRNA or siCTTN-2 and analyzed 24 hrs post-transfection. Cytoplasmic and nuclear fractions were subjected to immunoblot analysis for MRTFA, SRF, YAP1, pYAP1 (phosphorylated YAP1), and CTTN expression. For MRTFA, different exposure times were used to account for its varied distribution between cytoplasmic and nuclear compartments. α-Tubulin and lamin B2 served as cytoplasmic and nuclear markers, respectively. β-actin was used as a loading control.

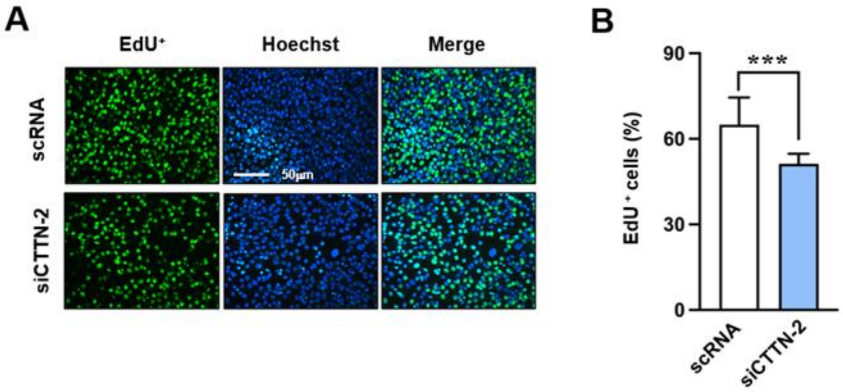

Figure S4. siCTTN-2 decreased EdU incorporation. C2C12 myoblasts were transfected with either control scRNA or siCTTN-2 and analyzed 24 hrs post-transfection. (**A**) Cell proliferation was evaluated by EdU incorporation (green) to label replicating cells, with Hoechst 33342 (blue) as a nuclear counterstain. Scale bar: 50 µm. (**B**) The percentage of EdU-positive cells was quantified using ImageJ software. Data are presented as means ± SEM (n = 3), with asterisks indicating statistical significance (***P < 0.001).

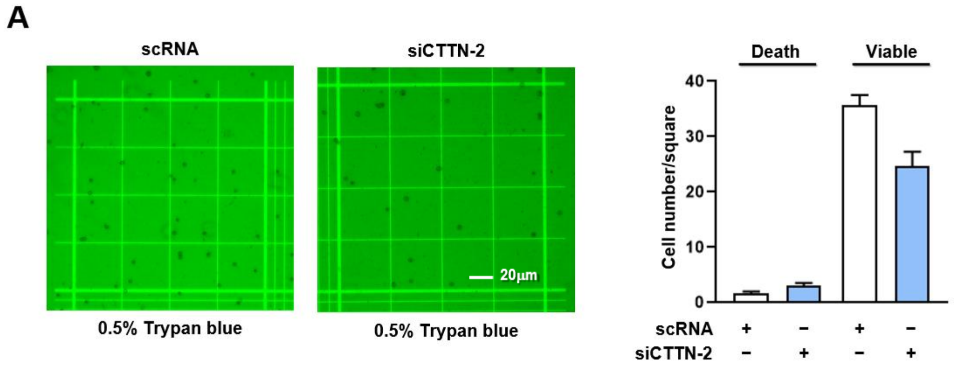

Figure S5. **siCTTN-2 reduced cell proliferation without inducing cell death.** C2C12 myoblasts were transfected with either control scRNA or siCTTN-2 and analyzed 24 hrs post-transfection. (**A**) Cells were harvested using Trypsin/EDTA and stained with 0.5% Trypan blue. Trypan blue stained cells indicated death cells. Scale bar: 20 μm.

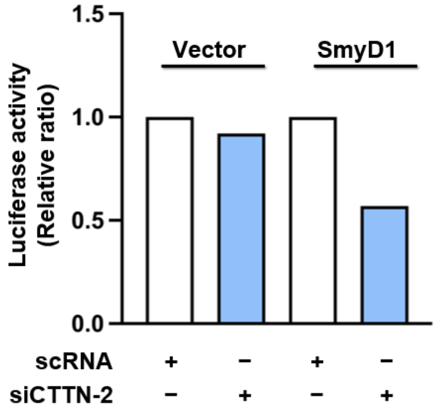

Figure S6. siCTTN-2 suppressed SRF transcriptional activity. C2C12 myoblasts were transfected with either the pGL3 vector (Vector) or pGL3 containing the SMYD1 promoter (SMYD1) along with control scRNA or siCTTN-2. Relative luciferase activity was measured 24 hrs post-transfection.

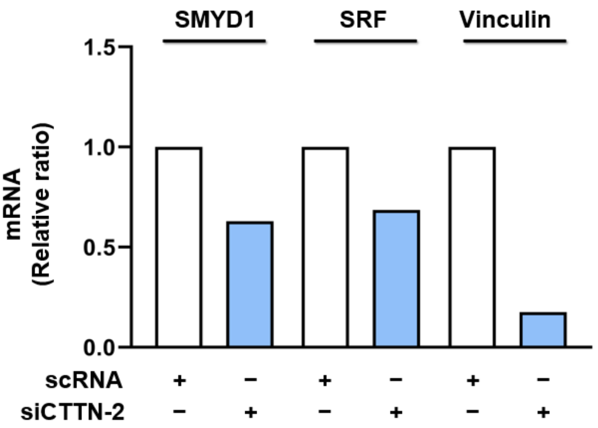

Figure S7. siCTTN-2 suppressed the mRNA levels of SRF-target genesSRF transcriptional activity. C2C12 myoblasts were transfected with either control scRNA or siCTTN-2, and mRNA levels of SRF, Vinculin, and SMYD1 were assessed by RT-*q*PCR, normalized to GAPDH expression 24 hrs post-transfection.

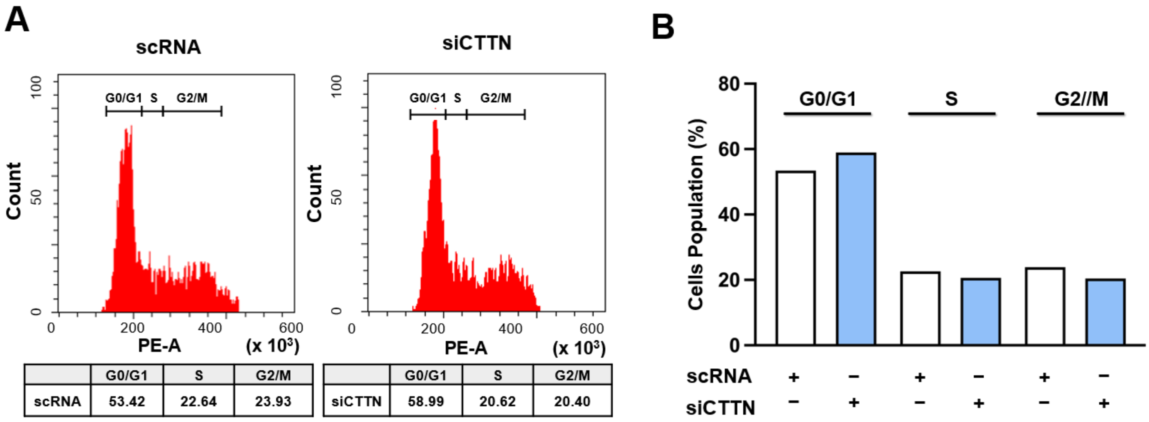

	G0/G1	S	G2/M
scRNA	53.42	22.64	23.93

	G0/G1	S	G2/M
siCTTN	58.99	20.62	20.40

Figure S8. siCTTN-2 impeded cell proliferation and cell cycle progression. (**A**, **B**) C2C12 myoblasts were transfected with either control scRNA or siCTTN-2 and analyzed 24 hrs post-transfection. Cell cycle analysis was performed using flow cytometry with scatter plots.
