# Supplementary material for: Essential Role of Cortactin in Myogenic Differentiation: Regulating Actin Dynamics and Myocardin-Related Transcription Factor A-Serum Response Factor (MRTFA-SRF) Signaling
Source: Int J Mol Sci. 2024 Dec 18;25(24):13564. doi: 10.3390/ijms252413564 (PMC11677934; doi:10.3390/ijms252413564)
Supplement: Supplementary file 1 [file ijms-25-13564-s001.zip › Supplementary Table S1-4.pdf]

Table S1. Oligonucleotide sequences for transfection

Gene	Oligonucleotide sequence (5´-3´)
scRNA (control RNA)	UCACAACCUCCUAGAAAGAGUAGA
siCTTN-1	CAGGAUAGGAUGGACAGAU
siCTTN-2	GACUGAGAAGCAUGCCUCC

Table S2. Primer lists and conditions for *q*RT-PCR

Gene	Primer sequence (5´-3´)		Product size	Annealing Temperature	Concentration		Cycle
					cDNA	Primer	
GAPDH	F.P	AACATCAAATGGGGTGAGGCC	262	58	2 ng/µl	0.5 µM	40
	R.P	GTTGTCATGGATGACCTTGGC					
PCNA	F.P	GAACCTGCAGAGCATGGACTC	201	58			
	R.P	GGTGTCTGCATTATCTTCAGCCC					
Cyclin D1	F.P	ACCAATCTCCTCAACGACCG	228	58			
	R.P	ACGGAAGGGAAGAGAAGGG					
Cyclin B1	F.P	GAGCTATCCTCATTGACTGG	125	58			
	R.P	CATCTTCTTGGGCACACAAC					
SRF	F.P	CTACACGACCTTCAGCAAGAG	141	58			
	R.P	GTATACACATGGCCTGTCTCAC					
Vinculin	F.P	ACCTGCAGACCAAAACCAAC	143	58			
	R.P	CTTACCGACTCCACGGTCAT					
SMYD1	F.P	GCATCTTCCCCAACCTGGGCCT	129	58			
	R.P	GGGCCCGGAGCTCAATCCTCAT					

Table S3. Primer lists for promotor cloning

Gene	Primer sequence (5´-3´)		Product size	Annealing Temperature	Concentration		Cycle
					cDNA	Primer	
SMYD1 promoter (mouse)	F.P	AAACTCGAGTTAGCAGAATCCGCAAG	969	58	2 ng/μl	0.5 μM	40
	R.P	AAAAAGCTTTCAGAGTCTGTCAGGCC					

Table S4. Antibodies list

Antibody	Type	Targeted species	Manufacturer	Cat. No.	Dilution ratio*
CTTN	Monoclonal	Rabbit	Cell Signaling Technology, Danvers, MA, USA	3503S	1:5,000
MyHC	Monoclonal	Mouse	DSHB, Iowa, IA, USA	MF20	1:1,000
MyoD	Monoclonal	Mouse	Santa Cruz Biotechnology, Dallas, TX, USA	sc-377460	1:1,000
MyoG	Monoclonal	Mouse	Santa Cruz Biotechnology, Dallas, TX, USA	sc-12732	1:1,000
SRF	Monoclonal	Rabbit	Cell Signaling Technology, Danvers, MA, USA	D71A9	1:10,000
MRTFA	Polyclonal	Rabbit	ABclonal, Woburn, MA, USA	A8504	1:10,000
YAP1	Monoclonal	Rabbit	Cell Signaling Technology, Danvers, MA, USA	14074S	1:10,000
pYAP1	Polyclonal	Mouse	Cell Signaling Technology, Danvers, MA, USA	4911S	1:10,000
Lamin B2	Monoclonal	Rabbit	Abcam, Cambridge, United Kingdom	ab151735	1:2,500
α-Tubulin	Monoclonal	Mouse	DSHB, Iowa, IA, USA	12G10	1:2,000
β-Actin	Monoclonal	Rabbit	Sigma-Aldrich Chemical, St. Louis, USA	A2066	1:10,000
Antibodies HRP-linked anti-rabbit IgG			Cell Signaling Technology, Danvers, MA, USA	#7074	1:10,000
Goat anti-mouse(H+L)			Thermofisher Sci., Waltham, MA, USA	#32430	1:2,000

*All blots were visualized using a TOPview ECL Femto (Enzynomics).
